# An integrated multidisciplinary model describing initiation of cancer and the Warburg hypothesis

**DOI:** 10.1186/1742-4682-10-39

**Published:** 2013-06-10

**Authors:** Edward A Rietman, Douglas E Friesen, Philip Hahnfeldt, Robert Gatenby, Lynn Hlatky, Jack A Tuszynski

**Affiliations:** 1 Center of Cancer Systems Biology, GeneSys Research Institute, Tufts University School of Medicine, Boston, 02142, USA; 2 Department of Oncology, Faculty of Medicine and Dentistry, University of Alberta, Edmonton, Alberta, T6G 1Z2, Canada; 3 H. Lee Moffitt Cancer Center & Research Institute, 12902 Magnolia Drive Tampa, FL, 33612, USA; 4 Department of Physics, University of Alberta, Edmonton, Alberta, T6G 2E1, Canada

**Keywords:** Warburg effect, Rayleigh-Benard convection, Aneuploidy, Glycolysis, Genomic instability

## Abstract

**Background:**

In this paper we propose a chemical physics mechanism for the initiation of the glycolytic switch commonly known as the Warburg hypothesis, whereby glycolytic activity terminating in lactate continues even in well-oxygenated cells. We show that this may result in cancer via mitotic failure, recasting the current conception of the Warburg effect as a metabolic dysregulation consequent to cancer, to a biophysical defect that may contribute to cancer initiation.

**Model:**

Our model is based on analogs of thermodynamic concepts that tie non-equilibrium fluid dynamics ultimately to metabolic imbalance, disrupted microtubule dynamics, and finally, genomic instability, from which cancers can arise. Specifically, we discuss how an analog of non-equilibrium Rayleigh-Benard convection can result in glycolytic oscillations and cause a cell to become locked into a higher-entropy state characteristic of cancer.

**Conclusions:**

A quantitative model is presented that attributes the well-known Warburg effect to a biophysical mechanism driven by a convective disturbance in the cell. Contrary to current understanding, this effect may precipitate cancer development, rather than follow from it, providing new insights into carcinogenesis, cancer treatment, and prevention.

## Background

The metabolic shift from aerobic to anaerobic glucose biochemical energy processing by cells is strongly correlated with the transition to cancer, or as some have come to characterize the process, a reversion to a more primitive and competitive level of cellular existence (Warburg [1], Szent-Gyorgyi [2]), which may still possess some rudimentary cooperative elements e.g. early metazoans (Davies and Lineweaver [3]). Our focus in this manuscript is to develop a molecular physics model based on non-equilibrium thermodynamics to quantitatively describe that process. By better understanding this transition we should be able to not only address cancer more effectively but also other metabolic diseases including mitochondrial diseases (e.g. [4]) and diseases of proton pumps (e.g. [5]). This modeling approach may also shed some light on the relationship between the Warburg effect for cancer and the so-called inverse-Warburg effect [6] for neurological diseases e.g. Alzheimer’s disease [7].

The living cell is an extremely complex molecular network of tens of thousands of different types of molecules from ionic species and small molecules to large polymers and polymer networks. Naturally, the number of large, multi-nanometer size polymers is not as high as the smaller molecules, but a shift in their numbers can easily result in the emergence of a disease state. For example, variation in the number of genes expressed due to changes in the genome (aneuploidy), can indicate cancer. Many of these large polymers are enzymes, or chemical reaction catalysts. Typical molecular reactions in the cell are represented by the relation:

(1) $$E+S\begin{array}{c} \overset{\mathrm{kf}}{\to }\\ \overset{\mathrm{kr}}{\leftarrow } \end{array}\mathrm{ES}\overset{k}{\to }E+P$$

where the symbols E, S, ES, P represent the enzyme, substrate, enzyme-substrate complex, and reaction product, respectively. The coefficients labeled by k’s represent forward, reverse and enzyme-substrate decomposition rate constants as indicated by their subscripts. Obviously, if there is a huge abundance of S and limited amount of E, the reaction is rate limited by the concentration of E. But if the cell is malfunctioning and producing an excess of E when an abundance of S is present, then the cell will increase the P concentration by massively parallel reactions. This is governed by the well-known Michaelis-Menten reaction kinetics of saturable chemical reactions [8]. This effect is also described by the Le Chatelier principle of reaction dynamics [9]. It states that chemical reactions move forward or backward so as to reduce excesses in the quantity of reactants or products, respectively, introduced into in the reaction vicinity.

**Figure 1:**
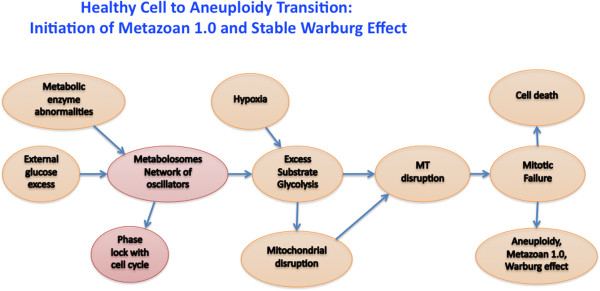
**Diagram of proposed transition from healthy to aneuploidy cell.** An excess of external glucose causes a series of phase transitions starting with glycolytic oscillators which induce a pH imbalance precipitating in mitochondrial membrane breakage followed by microtubule depolymerization and mitotic failure.

Given the reaction dynamics afforded by Le Chatelier’s principle, it is possible to imagine that an external concentration of, for example, glucose allowed entry into a cell would ripple through the molecular network of a cell and produce an excess of the appropriate glycolytic enzymes and other molecules associated with anaerobic processing. This would in turn produce an excess of lactic acid and a shift in the hydrogen ion concentration. Neither of these causes will necessarily induce a cell to transition to a higher entropy state (disorganization) of a cancer cell [10], but we argue that these reaction processes can disrupt the mitochondria and/or the cytoskeleton in part via the microtubule growth rate which is dependent on the pH value [11], a parameter known to change between normal and cancer cells. In particular, in cancer cells, intracellular pH is alkalized [12-15], and extracellular pH is acidified [12,14,15]. These biophysical changes in turn could contribute to mitotic failure and in rare cases aneuploidy – producing a stable cell exhibiting a glycolytic shift, i.e. the Warburg effect. Our hypothesis is schematically outlined in Figure 1.

## The model

We argue that non-equilibrium thermodynamics is a driving force for the ultimate transition of a cell from the normal to the cancer state, and that the first step in the process is an increased operation of the cytoplasmic glucose processing machinery due to a non-equilibrium mechanism analogous to Rayleigh-Benard convection (Figure 1) [16,17]. Our proposed scheme that describes the development of the cancer phenotype at a cellular level is essentially a biophysics model, which should clearly be considered in parallel with more biochemical schemes [18-22]. We begin by reviewing some relevant physics of nonlinear dynamics and thermodynamics.

### Rayleigh-Benard Convection

**Figure 2:**
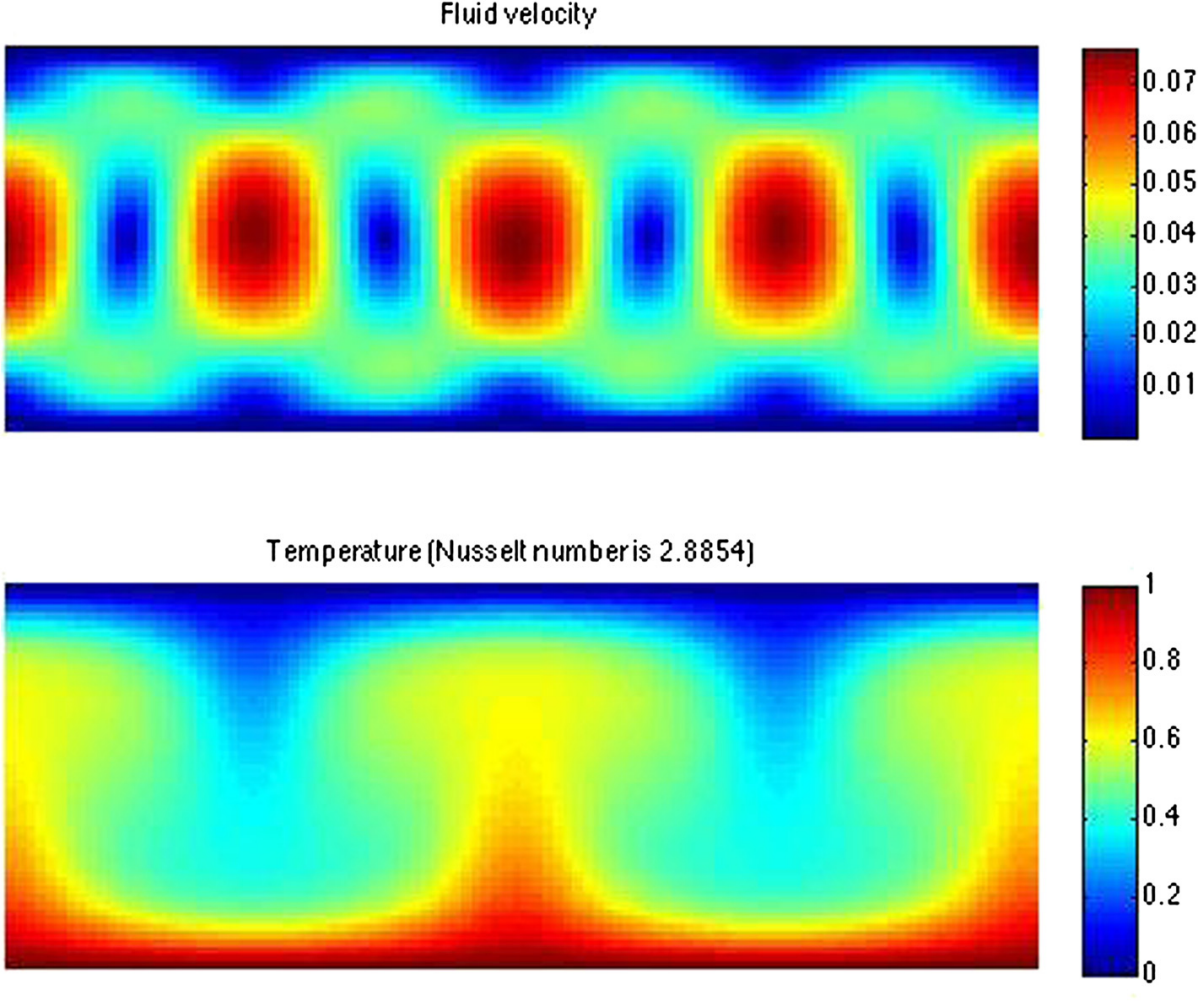
**Example of Rayleigh-Benard convection rolls.** (Based on GNU Matlab code of Parmigiani et al. [24]).

To appreciate the relevant dynamics, we can consider a thin layer of oil, of thickness *d*, lying on top of a uniformly heated metal plate. On top of the oil is another plate of high heat conductivity. The apparatus has non-thermally conducting bounding walls to keep the oil confined. We thus have a thermal gradient from a hot zone to a cooler zone. Initially the heat transfer is strictly via conduction from the hot surface to the cooler surface. The upper plate is kept at temperature *T*_*o*_ and the lower plate is kept at *T*_*o*_ + ΔT (ΔT > 0). As long as the temperature difference remains small, frictional forces in the fluid, due to viscosity, will keep convective motion near zero. Nonetheless, the conduction results in a small upward vertical vector from the bottom layer, and due to gravity a small downward vertical vector from the top layer. As a result of this, there will be a phase transition leading to a thermal convection mode of transport and an ordered state. The characteristic time for the displacement of the small regions of the fluid is given by:

(2) $$\tau_{\mathrm{RB}}\approx \frac{\mu }{\mathrm{\rho g\alpha d}\left(\mathrm{\Delta T}\right)}$$

where *μ*is the dynamic viscosity of the fluid, *α* is the thermal expansion coefficient of the fluid, *ρ* is the mean fluid density, and *g* is the gravitational acceleration. The condition for sustained convection is given by:

(3) $$\frac{\mathrm{\rho g\alpha}d^{3}}{\mu D_{T}}\left(\mathrm{\Delta T}\right)\geq R_{\mathrm{RB}}$$

where *D*_*T*_ is the thermal diffusion coefficient and the dimensionless constant *R*_*RB*_ is known as the Rayleigh number. In this case we have used the subscript *RB* to remind us that these are the Rayleigh-Benard relations. The Rayleigh number is related to the temperature differential. Above this convection threshold, the fluid undergoes a phase transition to an ordered phase – an example of a so-called symmetry breaking phenomenon characteristic of many critical phenomena in physics and other fields [23]. What is special about this example is that it is a non-equilibrium effect driven by the thermal gradient across the sample. At this point one can observe convection patterns known as *Rayleigh-Benard convection rolls* shown in Figure 2. These patterns are an observable manifestation of this non-equilibrium phase transition with an associated symmetry-breaking effect being the spatial periodicity of the rolls and a characteristic time scale representing an oscillation period.

Davies, Demetrius and Tuszynski [10] provided a qualitative argument that cancer is a dynamical phase transition. Here, we argue, by analogy, that a similar phenomenon to Rayleigh-Benard (R-B) convection, a symmetry breaking non-equilibrium phase transition could occur for cells grown with an excess glucose gradient between the outside of the cell and the inside of the cell, or with a critical change in the internal glycolytic enzyme distribution. This could represent a pre-cancerous transformation in terms of biological consequences of this physical process. Analogous to the R-B characteristic time relation above, we may now propose that for a cell:

(4) $$\tau \approx \frac{\nu }{\Phi \alpha \left(\mathrm{\Delta C}\right)}$$

where ΔC is the change in concentration, and using *ν* to represent the kinematic viscosity which has the units cm^2^ sec^-1^. Water has a viscosity of about 1 cP (cgs units) at ambient conditions. We will assume the viscosity inside a cell is about 1000 times higher (e.g. 10 P, Kalwarczyk et al. [25]). Instead of the force of gravity that has no appreciable effect on living cells at this scale, we will use energy density with units erg g^-1^ (with dimension cm^2^/sec^2^, instead of gravity’s cm/sec^2^), and represent it by the symbol, Φ. A similar parameter, energy-density-rate (erg g^-1^ sec^-1^ ) has been described by Chaisson [26] who discusses at length the application of energy-density-rate for multiple phenomena in the universe including evolution of life and evolution of stars. The parameter Φ is a universal parameter used to describe energy and the rate of energy flow; in other words, a specific metabolic rate, which represents the rate of energy transduction per unit mass. It is essentially a measure of the complexity of an open system *and* its rate of free energy utilization. In the case of small resting animals the value is on the order of 10,000 erg sec^-1^ g^-1^. For a single cell it is on the order of a few hundred erg sec^-1^ g^-1^. Obviously, it is a nonlinear function due to its relationship to metabolism. As has been well-known for many decades, metabolic rates scale with a fractional exponent in the range of 0.5 to 1.0, typically 0.75 [27]. For specific metabolic rates (metabolic rates per mass unit), these exponents become in the range from −0.5 to 0.0 [28]. Finally, we use α, which is the analog of the thermal expansion coefficient and therefore in this case the inverse of concentration, and we judiciously set its value to be 10^-4^. Keeping in mind it has the same units as inverse concentration but when multiplied with the concentration, as shown in Equation (4) the units cancel. We thus have the following units for Equation (4):

(5) $$T=\frac{L^{2}}{T}\begin{array}{cc} \frac{ML^{2}}{T^{2}} & \frac{1}{M} \end{array}=\frac{L^{2}}{T}\frac{T^{2}M}{ML^{2}}$$

Most of the remainder of the paper will be devoted to present arguments and analyses aimed at supporting the use of Equation (4) in the context of the Warburg effect.

**Figure 3:**
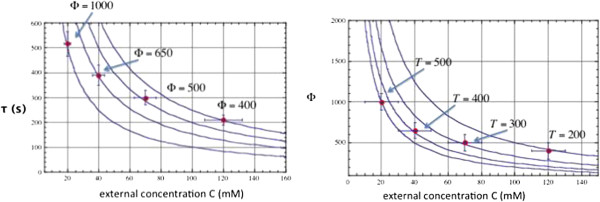
**Data from Table "Range of Glycolytic Oscillations in Yeast Extract" in Hess et al.**[29]**with ~10% error bars.** Curves showing oscillation time as a function of external concentration using Eq [4] and setting α = 10^-4^.

As can be seen in Figure 3, an increase in the external concentration results in the time constant falling more rapidly and larger values of the energy-density also result in smaller time constants. As the concentration increases, the value for Φ decreases. As the cell transitions from oxidative phosphorylation to glycolytic processing, the complexity of the molecular network transitions from mitochondrial to substrate level processing. The first primitive cells were anaerobic and had a lower Φ than later cells with mitochondria. Therefore, the model suggests that mitochondria are not being heavily exploited at this stage in the glycolytic oscillation experiment described by Hess et al. [29].

### Glycolytic oscillators

Several *in vitro* and *in vivo* experiments have already demonstrated that an increase in extracellular glucose [30,31] or lactic acid [32] concentration can lead to increased tumorigenesis. Further, hypoxia has been linked to causing the metabolic shift to glycolysis, and to cause malignant progression, with much experimental evidence [33-38].

Goldbeter [39] discusses at length glycolytic oscillations, and uses a series of differential equations to model the dynamics for some of the chemical species involved in the reactions. The observed oscillations come about as a result of the fact that there is a delay in manufacture of the intermediate nicotinamide adenine dinucleotide (NADH) and the fact that there is a finite number of molecular components available for the glycolytic processing. The cycle time has been observed in *S. carsbegensis* to be about 5 minutes using fluorescence of the glycolytic intermediate, NADH [40]. More interestingly, Hess et al. [29] measured the oscillation frequency for different doses of fructose or glucose as input. As shown in Figure 3, as the concentration of the glucose (or fructose) increases, the period of oscillation decreases. This can only come about from increased concentration in the number of enzymes to participate in the reactions. As the number of enzymes and the number of molecular components increase, so does the entropy because the increase in numbers enables more ways to dissipate free energy, in this case represented as chemical potential of a glucose gradient [41]. As the concentration increases the cycle time decreases indicating a more efficient processing of glucose per time unit.

More recently, Aromolaran et al. [42] describe, and show experimentally, glycolytically generated adenosine triphosphate (ATP) and Ca^2+^ waves propagating through a cell from application of glycolytic inhibitors focally injected from a glass pipette with a 1.5 micron diameter tip. The authors discover that glycolytically generated ATP is likely a key modulator of Ca^2+^ homeostasis. Of course, this has direct effect on the permeability of the mitochondrial wall, as shown by Yang et al. [43] who describe glycolytic oscillation depolarizing the mitochondrial membrane. The authors describe a model based on the logistic function showing there is a region where the oscillations are “too rapid for observation.” Though they do not use the term ‘chaos’, this is likely a chaotic state observed in the logistic functions and other “chaotic dynamical functions.”

In the case of Rayleigh-Benard convection rolls, as shown in Figure 2, the rolls can be modeled with a sine-circle map, for example, *θ*_*i*+1_ = *f*(*θ*_*i*_) where the function is periodic in the angle [44,45]. There are some theoretical arguments for the glucose oscillators being embedded in the cell membrane. Demetrius et al. [27] argue that the enzymes’ concentration would oscillate due to periodicities in the redox potential and the result could be modeled as harmonic oscillators. Further, Tyner et al. [46] measure electrical gradients in the cell, and we show that a relevant protein, glyceraldehyde 3-phosphate dehydrogenase (GAPDH) associated with glucose processing in the cytoplasm accumulate at the membrane thus showing experimental support for our hypothesis that molecular oscillators accumulate at boundaries. Lastly, Pokorny [47] suggests Duffing oscillators [48] as a potential model of oscillatory states of a cell. In the following subsections we first provide some experimental validation for the differences in GAPDH localization. Then we follow that with some simplistic analytical modeling to show phase locking of oscillators potentially resulting chaos and disruptions of mitochondria.

### Experimental validation for GAPDH distribution

These predictions of GAPDH distribution were tested by using antibodies against GAPDH in normal human mammary epithelial cells (HMECs) under normal culture conditions, purchased from Life technologies.

**Figure 4:**
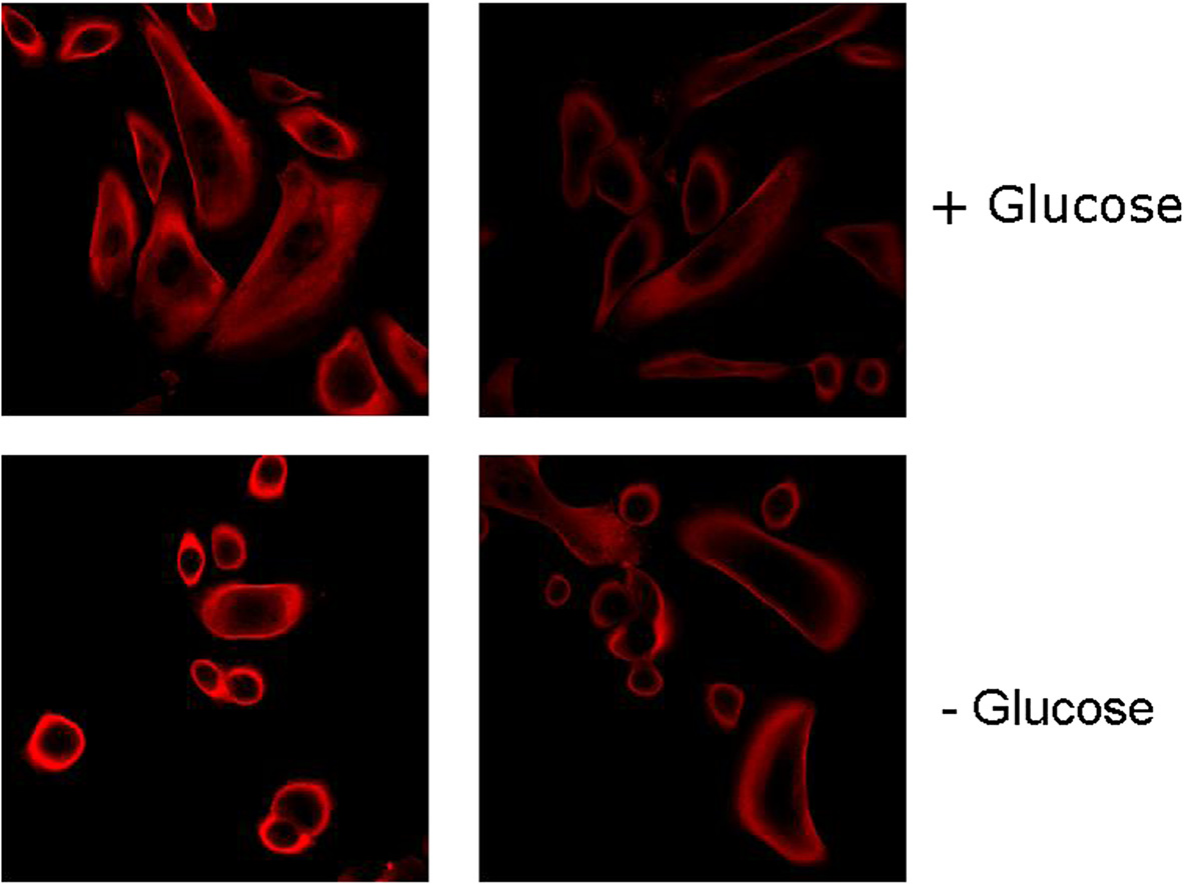
Intracellular localization of GAPDH in HMEC cells on glass slides with and without glucose.

Briefly, HMEC cells were grown on coverslips and incubated at 37 C, 5% CO_2_ until they reached 50% confluency. They were then fixed with 4% paraformaldehyde, permeabilized with 0.1% Triton and blocked for 1 hour in 1%BSA/1X PBS. The cells were then incubated with 1º Antibody (GAPDH from Santa Cruz #sc-25778) at 1:100 for 3 hours at room temperature. After 3 washes with 1X PBS, cells were then incubated with 2º Antibody (Alexa Fluor 647 anti-rabbit from Invitrogen A21244) for 2 hours at room temperature. After 3 washes in 1X PBS, some cells were incubated for 5 minutes with a dilute solution (300 nm in PBS) of with 4',6-diamidino-2-phenylindole (DAPI) which is a fluorescent stain that binds strongly to A-T rich regions in DNA and used to demarcate the nucleus. After 3 washes with 1X PBS, coverslips were mounted on slides with Prolong Gold (Invitrogen P36934). Cells were then imaged using the Leica SP5 Confocal Microscope. As shown in Figure 4, intracytoplasmic GAPDH was present in highest concentrations in the cell periphery and virtually absent from the perinuclear regions of the cytoplasm.

**Figure 5:**
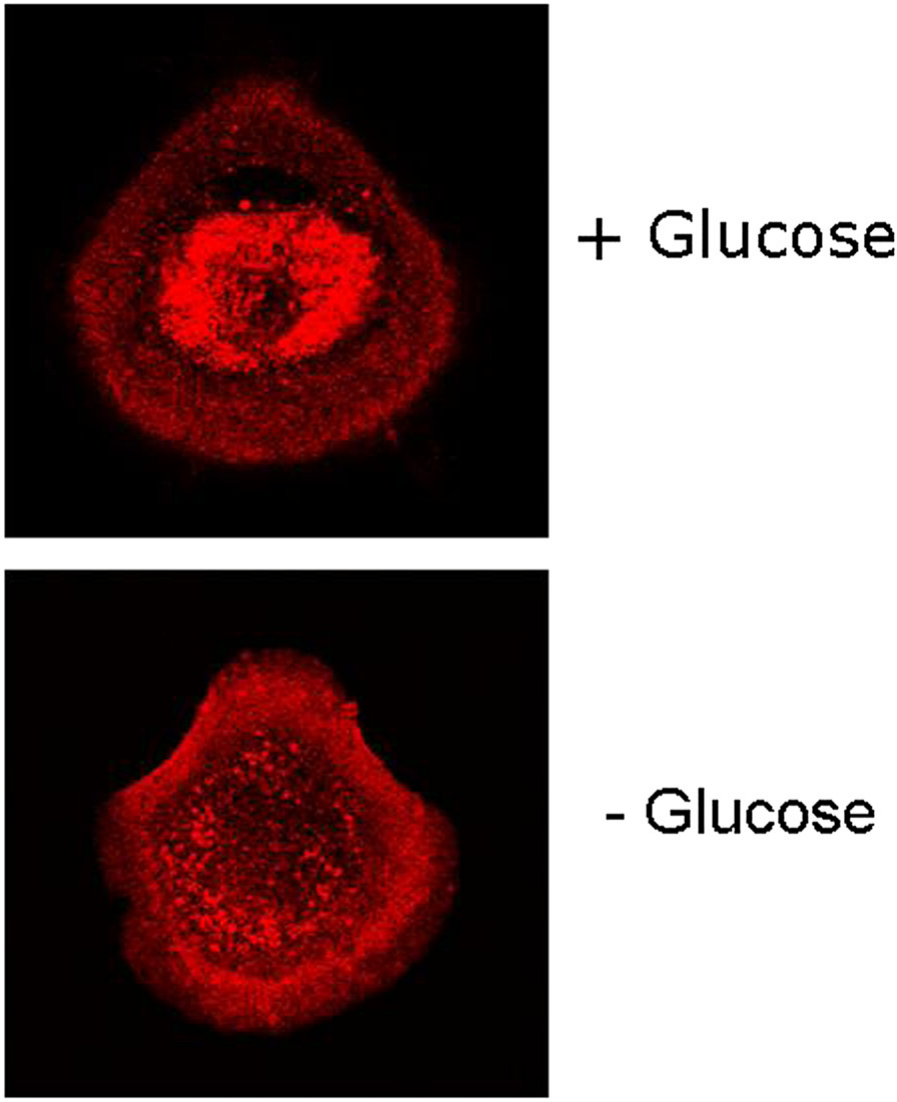
Intracellular localization of GAPDH in HMEC cells on Cytoo Chips with and without glucose.

In some experiments HMEC cells were seeded on glass slides or Cytoo Chips at a concentration of 30 K cells/ml and incubated overnight at 37 C. The next day, cells were glucose starved for 1 hour. Control cells were incubated in complete media. After 1 hour, cells were washed in 1X PBS and fixed for 10 minutes in 4% Paraformaldehyde, permeabilized in 0.1% Triton for 10 min, and blocked in 1%BSA/PBS for 1 hour. For the immunostaining procedure, cells were incubated in a 1:100 dilution of GAPDH at room temperature for 1 hour, followed by incubation with the secondary rabbit antibody, Alexa Fluor 594, at 1:500 for 30 minutes at room temperature. The cells were imaged with a Zeiss Confocal Microscope. Figure 4 shows intracellular localization of GAPDH in HMEC cells on glass slides with and without glucose while Figure 5 shows intracellular localization of GAPDH in HMEC cells on Cytoo Chips with and without glucose.

These clear experimental results support our conjecture that there is an increase in the glycolytic processing as a result of an external glucose gradient. We now describe some analytical models outlining the phase locking of the glucose oscillators and the implications.

### Sine circle Map modeling

As suggested in the above experimental work the glucose oscillators likely accumulate at specific regions in the cell to make maximum use of the large glucose gradient. To model this phenomena, we will use an array of sine-circle maps in a grid, called a coupled map lattice, with a global coupling parameter representing the external glucose concentration. This global coupling will result in increases in oscillation as the coupling constant increases. The sine-circle map, represented as a difference equation, is given by

(6) $$\theta_{t+1}\leftarrow \theta_{t}+\frac{\kappa }{2\pi }\sin\left(2\pi \theta_{t}\right)$$

**Figure 6:**
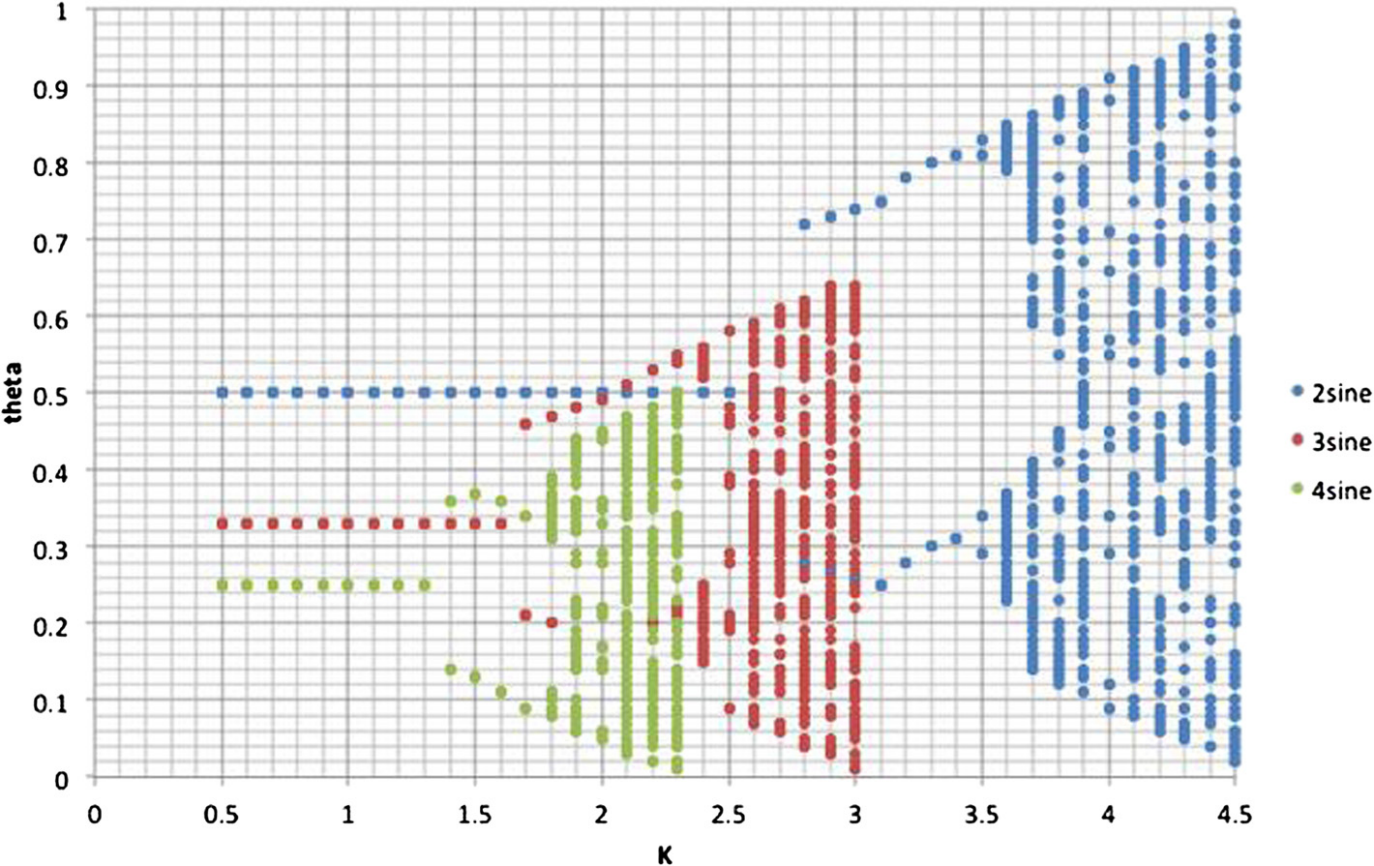
Bifurcation diagram for sine-circle map. Computed after 10,000 iterations.

As κ increases from 0.5 to 4.5 the output of the function transitions from a fixed point to 2-cycle, 4-cycle and finally chaos. This is a typical bifurcation diagram similar to that produced by the logistic function [49]. If we replace the 2 in the argument of the sine function with 3 or 4 it simply moves the bifurcation points on the *θ****–****κ* curve. This is shown in Figure 6.

There has been research showing a quasi-periodic route to chaos in biochemical systems [21,39,50,51]. The biochemical system, in these cases, glycolysis, starts at some given frequency and as external glucose is pulsed, starting at a frequency similar to the glycolysis value, the biochemical reactions begin to phase lock with the external pulses and first results in increased amplitude followed by period doubling as the external glucose pulse frequency increases. Finally, at a stochastic external glucose pulse frequency, the observed internal pulses are only about 3–4 X higher than the original glucose oscillations, but the actual sine wave appears as if there is a frequency and/or amplitude modulation. The oscillations observed in the cell extracts are not chaotic. Analytical models of these biochemical systems [52,53], based on differential equations, support a periodic-doubling route to chaos, though in the real biochemical systems chaos is rarely observed. This is likely due to the fact that a cell is not just “a bag of chemicals” but really a hybrid between a chemical network and molecular nanomachines. The equations of dynamics also apply to mechanical systems, yet real machines are not capable of being driven into chaotic state, because of friction and physical constraints in the machine. Similarly, nanomachines are not likely able to be driven into chaotic states because of secondary bonding effects, e.g. van der Waals forces, and steric hindrance and potential energy surfaces [54].

In the above, cell-free extract, experiments, the investigators monitored NADH to observe the oscillations in the range of 0.002 to 0.005 Hz. Yang, et al. [43] did experiments on tissue models of rabbit ventricular myocytes and observed adenosine diphosphate (ADP) oscillations. Of course, when combining these experimental observations it must be remembered that there are 2 NADH molecules and 4 molecules of ADP per glucose molecule in the glycolysis reaction. Yang et al. [43] observed oscillation increases from about 0.02 to 0.067 Hz; but like the cell-free experiments, they did not observe chaos. Since chaos has been observed in real-world chemical systems [55], the lack of observed chaos in these biochemical and tissue experiments can be explained as follows.

In the case of tissue-based experiments, the oscillators are operating at a fixed frequency all driven by chemical kinetics and Le Chatelier’s principle. The observation of increase in frequency is likely the mean-field effect from an increase in the *numbers* of oscillators and this would also account for the observed effect on decrease of energy-density-rate as the concentration increases. This means the cell is adapting to the excess external glucose by producing more glycolysis oscillators. As will be shown shortly, these oscillators can phase lock with each other and produce oscillations at a frequency about 2 or 3 times higher.

In the case of the cell-free extract, it is not likely that more oscillator-components are being produced on demand, so Le Chatelier’s principle will not be modulating the overall molecular network. Instead, the existing molecular components for oscillator construction are fixed, and more *in situ* oscillators may form because of the excess glucose. Again these oscillators can phase lock and produce the observed frequencies.

We can describe the phase locking with a coupled map lattice. Since the glucose oscillators are modeled here as a sine-circle map, we build our coupled map lattice from these. The definitive reference on coupled map lattices is by Kaneko [56]. Coupled map lattices (hereinafter CML and not to be confused with chronic myelogenous leukemia), are lattice models with, usually, difference equation mapping relations in the cells comprising the lattice. The cell updates are given by *x*(*i*) ← *f*(*x*(*i*)). And to include “diffusion” or coupling between the cells one typically modifies the update equation as

(7) $$x_{n+1}\left(i\right)\leftarrow \left(1-\epsilon \right)ƒ\left(x_{n}\left(i\right)\right)+\frac{\epsilon }{2}\left[ƒ\left(x_{n}\left(i+1\right)+ƒ\left(x_{n}\right)\left(i-1\right)\right)\right]$$

This is a one-dimensional CML, where the left and right neighbor of cell *i* are coupled to cell *i*. We use the sine circle map as the main function *ƒ*

(8) $$\theta_{t+1}\leftarrow \theta_{t}+\frac{\kappa }{2\pi }\sin\left(2\pi \theta_{t}\right)$$

Rather than use a global coupling parameter, ϵ, as is usually done, we assume a self-regulatory threshold dynamics. The adaptive mechanism is triggered when a glycolytic oscillator exceeds a critical threshold x*, and excess is passed on to its neighbor. As observed in spin-glasses [57], we assume a symmetry breaking effect, so that only one neighbor actually receives the excess and which neighbor, (e.g. left or right) is preserved throughout the dynamical update. Our algorithm for a one-dimensional array is thus:

1. for each oscillator compute the current state

$$x\left(i\right)\leftarrow x\left(i\right)\frac{k}{2\pi }\sin\left(2\mathrm{\pi x}\left(i\right)\right)$$

2. check threshold

$$\mathrm{if}\;x\left(i\right)>x*$$

$$\mathrm{delta}\left(i\right)=x\left(i\right)-x*$$

else

$$\mathrm{delta}\left(i\right)=0$$

3. send excess to neighbor

$$x\left(i+1\right)\leftarrow x(i+1)+\mathrm{delta}\left(i\right)$$

4. reset delta array

$$\mathrm{delta}\left(i\right)=0$$

**Figure 7:**
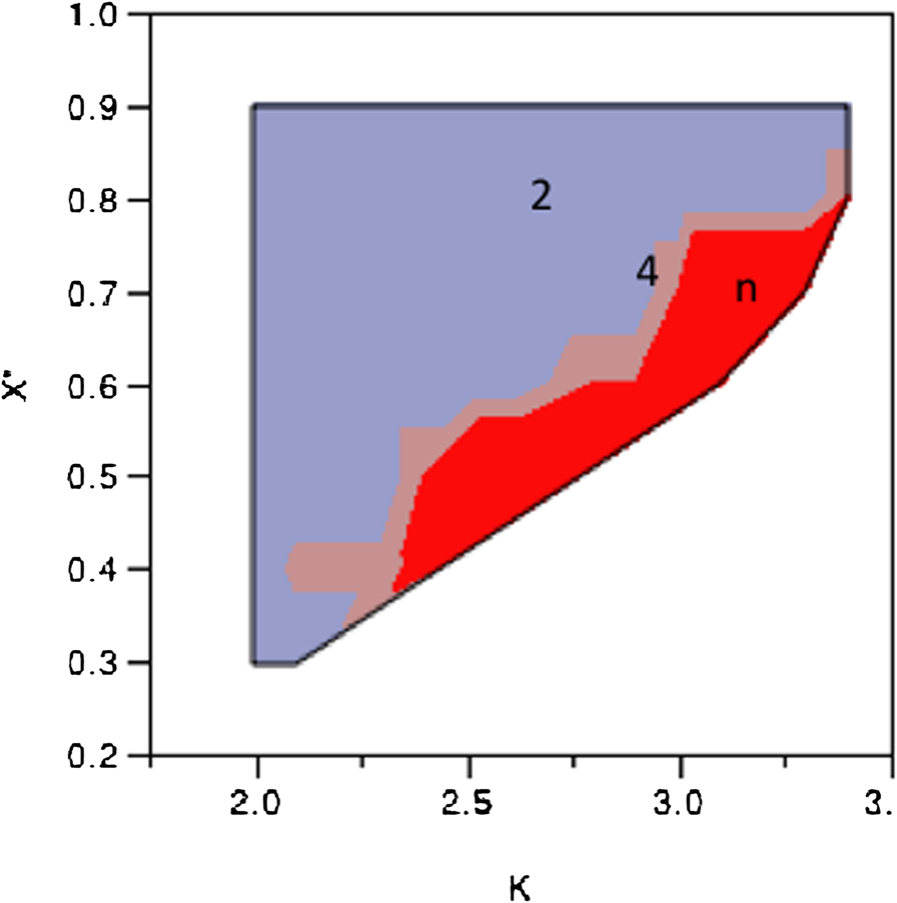
**Phase diagram for a coupled-map.** Numbers indicate cycle of oscillator. White regions are either undefined or represent a fixed point.

Hence, the adaption is triggered when x(i) > x*. This causes unidirectional transport *δ*(*i*) = *x*(*i*) − x* ; *x*(*i* + 1) = *x*(*i* + 1) + *δ*(*i*). This algorithm has been shown to be capable of universal computation [58]. It does, however require careful tuning of the threshold and bifurcation parameters. For example, since the values of the sine function can reach 1.0 and if x* = 0.15 and κ = 1.0, then the map can blow up. The full phase diagram for x* and κ is given in Figure 7. As expected for any chaotic attractor, there are regions of fixed point, complex oscillations (e.g. 2-cycle, 4-cycle) and regions we label as undefined because one or more oscillators blew up.

Kaneko [56] discusses many options of coupling the oscillators. In all cases the phase diagram contains regions of fixed points, oscillations, and undefined or chaos. This is to be expected from the bifurcation of the function as shown in Figure 6. The hard boundary in our phase diagram at κ = 2 is to be expected as shown in Figure 7. The fact that the oscillations begin prior to 2.5 is due to the fact that the thresholding and signal transfer changes the dynamics. The regions in the phase diagram labeled *n* are for n-cycle, or strange attractor [44].

In mapping this phase diagram to glycolytic oscillations we would not expect all threshold values to be valid. If we set the threshold to 0.5 then the bifurcation parameter represents the glucose dosage and we have 2 cycles over a small range until the 4-cycles followed by continued increase in the glucose (bifurcation parameter) results in n-cycles. A 4-cycle in this phase space would look like a doubled 2-cycle, amplitude modulated glycolytic oscillator; and an n-cycle would look like an amplitude or frequency modulated 2-cycle or 4-cycle. These types of modulations have been observed by Hess et al. [29] and von Klitzing and Betz [59].

### Mitochondrial transition

We now turn our attention to the mitochondria and explore the possible implications from glycolytic oscillations on mitochondrial stability. It is known that the glycolytic oscillations result in similar pH oscillations (e.g. Hess et al. [29]). But these oscillations are about π/2 out of phase. This has implications on the polarization of the mitochondrial membrane. The cell contains buffer mechanisms to minimize pH imbalance, but too much polarization of the mitochondrial membrane will cause the mitochondria to break down. The phase lag in pH can lead to a potential problem.

Galante et al. [20] showed that an increase of protons (decrease in pH) to an assay of heart mitochondria results in a decrease in the Michaelis-Menten rate constant *K*_*m*_, and an increase in the forward velocity, *V*_*f*_, of the reaction

(9) NADH+NADP⇔NAD+NADPH

The ratio of *V*_*f*_/*K*_*m*_ shows an actual phase transition at pH 7.5. The authors did not discuss the significance of this phase transition. Later work by Aromolaran et al. [42] showed waves of Ca^2+^ ions traversing the cell as a result of a localized ATP perturbation. These waves are able to traverse the entire cell within 30 seconds – far faster than diffusion. Other work by Ramanujand and Herman [60] show a nonlinear scaling of glucose metabolism in normal and cancer cells, where the scaling exponent is different for both types of cells. This is analogous to our observed Φ variation as a function of glucose. Lastly, Aon et al. [61] describe experiments on percolation and criticality [62] in mitochondrial networks of a cell. The authors used a local perturbation induced by a two-photon laser excitation. They observed a cell-wide transition to take place within 4 seconds resulting in depolarization of the majority of the mitochondria in the cell.

This depolarization of the mitochondria membrane also accompanies an ATP depletion. This can in turn effect the following reactions [63]:

•D-glucose + ATP → D-*glucose*-6-*phosphate* + ADP

•D-fructose-6-phosphate + ATP → D-*fructose*-1,6-*bisphosphate* + ADP

•1,3-bisphosphoglycerate + ADP → 3-*phosphoglycerate* + ATP

•phosphoenolpyruvate + ADP → *pyruvate* + ATP

with the following heats of formation, respectively:

$$\begin{array}{l} \mathrm{\Delta G}=-16.7\mathrm{kJ}/\mathrm{mole},\;\mathrm{\Delta G}=-14.2\mathrm{kJ}/\mathrm{mole},\;\\ \mathrm{\Delta G}=-18.8\mathrm{kJ}/\mathrm{mole},\;\mathrm{\Delta G}=-31.4\mathrm{kJ}/\mathrm{mole} \end{array}$$

ATP is needed to maintain Ca^2+^. A lag in production of ATP, as the above reactions compete with other reactants and products in the overall molecular network, could induce changes in the cytoskeleton via pH effects on the growth dynamics of the microtubules.

**Figure 8:**
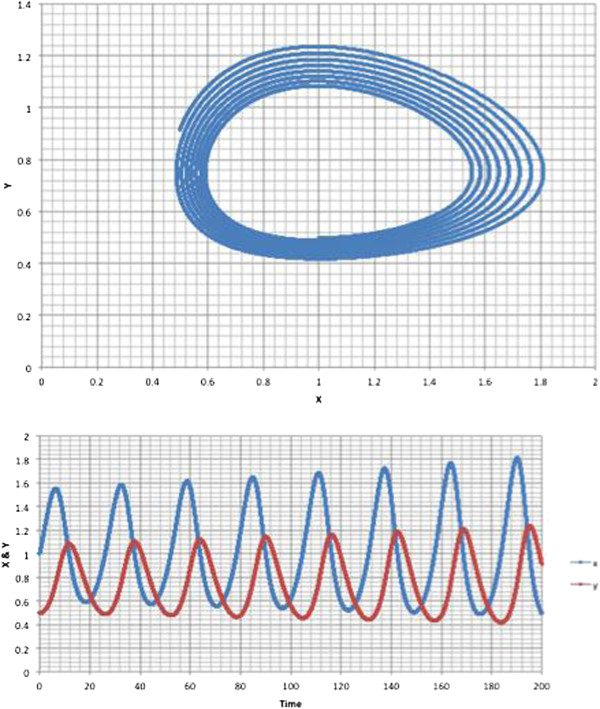
Example of dynamics for coupled system similar to glucose and pH oscillations in a cell a = 0.3, b = 0.4, c = 0.2, d = 0.2.

The dynamics of the entire system of substrate metabolism of glucose is beyond the scope of this paper and has already been reported in the literature [64-67]. But even a small system of two coupled components, where one is phase lagged with respect to the other can give rise to very complex dynamics. For example, the system given by:

(10) $$\begin{array}{c} \mathrm{dx}/\mathrm{dt}=\mathrm{ax}-\mathrm{bxy}\\ \mathrm{dy}/\mathrm{dt}=-\mathrm{cy}+\mathrm{dxy} \end{array}$$

can result in essentially positive feedback. We show this in Figure 8. Gehrmann et al. [68] model the complete glycolysis reaction system and shown that the enzyme, phosphofructokinase (PFK) is a key component for not only stabilizing ATP but key because it can also result in positive feedback. This in turn can result in a runaway oscillation system similar to that shown in Figure 6. The authors also show chaos, but that is likely an artifact of the modeling since real chemo-mechanical systems comprising nanoscale machines operating on molecules could not possibly result in chaos. More likely the system would break. In this case apoptosis would be the result, not chaos.

It is interesting to note that our analysis suggests that ATP oscillations could effect

2phosphoenolpyruvate+ADP→pyruvate+ATP

amongst other reactions. Using Le Chatelier’s principle we can argue that forcing this reaction in the reverse direction by using 3-bromopyruvate will deplete the ATP and thus induce the cancer cell to enter into an apoptotic state. This type of behavior has in fact been reported by Ko et al. [69] and Mathupala et al. [70], who reported significant reduction in tumor volume in mice by treatment with 3-bromopyruvate.

### Cytoskeleton disruption

The metabolic shift to glycolysis leads to acidosis, which subsequently results in an acidic extracellular pH [12]. Gillies has documented an acidic extracellular environment in numerous tumors [12]. Several studies link acidosis to genomic instability. Morita et al. show low pH leads to sister-chromatin exchanges and chromosomal aberrations [71,72]. Brusick, Cifone, and Cipollaro report that low pH environments (~pH 6.5) caused genomic toxicity [73-75]. Several studies link the tumor environment, with hypoxia and low pH as inducing genomic instability by DNA repair activity being reduced [76], and enrichment for mismatch-repair deficient cells [77].

Thus, convincing evidence exists that a hypoxic and acidic environment will lead to genomic instability due to impairment of DNA repair processes.

In addition, Suresh [78] has reviewed the biomechanics of normal and cancer cells and shows that transformed cells routinely have altered deformability. This effect is likely due to the differences in the actin microfilaments – the major structural element of a cell [79,80].

The cytoskeleton also consists of microtubules that have a bending stiffness 2.6 X 10^-23^ N m^2^ which is about 1000 times stiffer than actin filaments [78]. Microtubules, unless stabilized by ligands or microtubule-associated protein, are in a constant dynamic instability process being polymerized and depolymerized with a half-life of about 2 minutes [81]. This dynamic instability phenomenon has been mathematically modeled in detail by Sept et al. [82], and Bolterauer et al. [83] among others. The ends of actin filaments and microtubules are caped with ATP and guanosine triphosphate (GTP), respectively. GTP is created by the citric acid cycle in the mitochondria. Obviously, a mitochondria failure may reduce the concentration of ATP and GTP, if other systems do not compensate for the mitochondrial failure. A reduction in GTP results in a higher rate of depolymerization of microtubules [84] and a decrease in ATP concentration reduces the rate of growth of actin filaments [85]. Too few microtubules and/or microtubules being too short to participate in proper spindle pole formation can lead to mitotic catastrophe and/or potentially lead to chromosome instability. Bakhoum et al. [86] and Thompson et al. [87] describe the mechanism for this instability. Persistent mis-oriented attachment of chromosomes to the spindle microtubules leads to severe chromosome segregation defects.

## Conclusions

### Summary

We have described an integrated system model for the progression of a healthy cell to a cancer state and some of the implications. The potentially aberrant state of the cell may start by an excess glucose or other nutrient external to the cell impacting the cell or by internal defects leading to metabolic enzyme redistribution processes. This excess nutrient is essentially a chemical potential difference between inside and outside of the cell creating stress. Through a process analogous to Rayleigh-Benard convection, stable molecular oscillators accumulate in the cytoplasm to exploit this chemical gradient. The continued activity of these oscillators results in mitochondrial destabilization, which may occur as a non-equilibrium phase transition. Once the mitochondria begin to perform aberrantly there will be a chemical imbalance in key components for microtubule assembly/disassembly. This imbalance is driven by Le Chatelier’s principle. The disruption in microtubule lengths and/or microtubule count will lead to chromosomal instability via kinetochore-microtubule dynamics finally leading to mitotic failure. In unlucky cases this will result in chromosome mis-segregation and cancer if mitotic catastrophe does not occur.

### Clinical implications

The above synthesis of ideas surrounding the subject of Warburg initiation, or the transition from aerobic glycolysis to anaerobic suggests not only an avenue for treatment but also an avenue for prevention of cancer.

We hypothesize that excess of glucose and sugar-like energy sources or metabolic enzyme abnormalities, through a non-equilibrium phase transition (a symmetry breaking phenomenon) analogous to the Rayleigh-Benard convection, may cause a cell to prefer to process this energy source using substrate glycolysis. Continued excess substrate glycolysis will cause further phase transitions to disrupt the mitochondria via depolarization and also disrupt microtubule dynamics. When a cell then passes through mitosis, the chance of mitotic failure is increased. When a cell enters mitotic failure, it may undergo an aneuploidy event [88,89]. All this suggests that a low glycemic diet would lower the incidence of cancer, and may suggest a mechanism why metformin, which lowers blood glucose levels, is associated with improved outcomes in diabetic cancer patients [90,91] and reduced risk of pancreatic cancer [92].

The above synthesis of ideas also supports targeting cells that have made the glycolytic switch. For instance, the work of Pedersen [93] and his colleagues (Ko et al. [69,94]; Mathupala et al. [70]) have used 3-bromopyruvate to inhibit glyceraldehyde 3-phosphate dehydrogenase (GAPDH), which effectively inhibits glycolysis [95]. In addition, 3-bromopyruvate may force, via Le Chatelier’s principle, some reverse reactions to essentially deprive the cancer cell of substrate-created ATP. This leaves the cell little choice except to enter apoptosis. We further hypothesize that a Br derivative of 3-phosphoglycerate would similarly, though perhaps not as energetically, and perhaps not as toxically, facilitate via Le Chatelier’s principle, a reverse reaction to deprive a cancer cell of ATP.

Further, since microtubule dynamics are dysregulated by glucose oscillations and its associated pH oscillations, we speculate that metronomic dosing of microtubule poisons (e.g. nocodazol, taxol, vinblastine) would be an effective treatment strategy for cancers.

In our list of “prescriptions” above many of these are already known or in use. These current practices are essentially “prediction” of our theory and were strictly based on biophysics with little detailed biochemical or cellular biochemistry being considered.

## Competing interests

The authors declare no competing interests in this research.

## Authors’ contributions

EAR proposed to JAT using R-B convection as an analog. EAR, JAT, and DEF developed the integrated theory. EAR wrote the modeling software and conducted the simulations. RG contributed to experiments. PH and LH contributed insight into cancer and added further refinements of the integrated system model. All authors contributed to writing the manuscript. All authors read and approved the final manuscript.

## References

[B1] Warburg O On the origin of cancer cells Science 1956 123 309 314 10.1126/science.123.3191.309 13298683

[B2] Szent-Györgyi A The living state and cancer Proc Nat Acad Sci USA 1977 74 2844 2847 10.1073/pnas.74.7.2844 268635 PMC431314

[B3] Davies PCW Lineweaver CH Cancer tumors as Metazoa 1.0: tapping genes of ancient ancestors Phys Biol 2011 8 015001 10.1088/1478-3975/8/1/015001 21301065 PMC3148211

[B4] Miceli MV Jazwinski SM Common and cell type-specific responses of human cells to mitochondrial dysfunction Exp Cell Res 2005 302 270 280 10.1016/j.yexcr.2004.09.006 15561107

[B5] Marino ML Fais S Djavaheri-Mergny M Villa A Meschini S Lozupone F Venturi G Della Mina P Pattingre S Rivoltini L Proton pump inhibition induces autophagy as a survival mechanism following oxidative stress in human melanoma cells Cell death & disease 2010 1 e87 10.1038/cddis.2010.67 21368860 PMC3035900

[B6] Demetrius LA Simon DK An inverse-Warburg effect and the origin of Alzheimer’s disease Biogerontology 2012 13 583 594 10.1007/s10522-012-9403-6 23086530

[B7] Bennett DA Is there a link between cancer and Alzheimer disease? Neurology 2010 75 1216 1217 20922851

[B8] Kacser H Burns JA The control of flux Symp Soc Exp Biol 1973 27 65 104 4148886

[B9] Atkins PW Physical Chemistry 1986 New York: W. H. Freeman and Company

[B10] Davies PC Demetrius L Tuszynski JA Cancer as a dynamical phase transition Theor Biol Med Model 2011 8 30 10.1186/1742-4682-8-30 21867509 PMC3177875

[B11] Regula CS Pfeiffer JR Berlin RD Microtubule assembly and disassembly at alkaline pH J Cell Biol 1981 89 45 53 10.1083/jcb.89.1.45 7228899 PMC2111771

[B12] Gillies RJ Raghunand N Karczmar GS Bhujwalla ZM MRI of the tumor microenvironment J Magn Reson Imaging 2002 16 430 450 10.1002/jmri.10181 12353258

[B13] Reshkin SJ Bellizzi A Caldeira S Albarani V Malanchi I Poignee M Alunni-Fabbroni M Casavola V Tommasino M Na^+^/H^+^ exchanger-dependent intracellular alkalinization is an early event in malignant transformation and plays an essential role in the development of subsequent transformation-associated phenotypes FASEB journal : official publication of the Federation of American Societies for Experimental Biology 2000 14 2185 2197 10.1096/fj.00-0029com 11053239

[B14] Stock C Schwab A Protons make tumor cells move like clockwork Pflugers Archiv : European journal of physiology 2009 458 981 992 10.1007/s00424-009-0677-8 19437033

[B15] Harguindey S Arranz JL Wahl ML Orive G Reshkin SJ Proton transport inhibitors as potentially selective anticancer drugs Anticancer Res 2009 29 2127 2136 19528473

[B16] Bergé P Pomeau Y Vidal C Order within chaos: towards a deterministic approach to turbulence 1986 New York: Wiley

[B17] Tritton DJ Physical Fluid Dynamics 1977 New York: Van Nostrand Reinhold

[B18] Vander Heiden MG Locasale JW Swanson KD Sharfi H Evidence for an Alternative Glycolytic Pathway in Rapidly Proliferating Cells Science 2010 329 1492 1499 10.1126/science.1188015 20847263 PMC3030121

[B19] Yakovlev G Hirst J Transhydrogenation reactions catalyzed by mitochondrial NADH-ubiquinone oxidoreductase (Complex I) Biochemistry 2007 46 14250 14258 10.1021/bi7017915 18001142

[B20] Galante YM Lee Y Hatefi Y Effect of pH on the mitochondrial energy-linked and non-energy-linked transhydrogenation reactions J Biol Chem 1980 255 9641 9646 7430091

[B21] Termonia Y Ross J Oscillations and control features in glycolysis: numerical analysis of a comprehensive model Proc Nat Acad Sci USA 1981 78 2952 2956 10.1073/pnas.78.5.2952 6454892 PMC319477

[B22] Hanahan D Weinberg RA Hallmarks of cancer: the next generation Cell 2011 144 646 674 10.1016/j.cell.2011.02.013 21376230

[B23] Anderson PW Basic Notions Of Condensed Matter Physics 1997 Boulder: Westview Press

[B24] Parmigiani A Huber C Chopard B Latt J Bachmann O Application of the multi distribution function lattice Boltzmann approach to thermal flows The European Physical Journal Special Topics 2009 171 37 43 10.1140/epjst/e2009-01009-7

[B25] Kalwarczyk T Ziȩbacz N Bielejewska A Zaboklicka E Koynov K Szymański J Wilk A Patkowski A Gapiński J Butt H-J Hołyst R **Comparative Analysis of Viscosity of Complex Liquids and Cytoplasm of Mammalian Cells at the Nanoscale** Nano Letters 2011 11 2157 2163 10.1021/nl2008218 21513331

[B26] Chaisson EJ Cosmic Evolution: The Rise of Complexity in Nature 2001 Cambridge: Harvard University Press

[B27] Demetrius L Tuszynski JA Quantum metabolism explains the allometric scaling of metabolic rates Journal of the Royal Society Interface 2010 7 507 514 10.1098/rsif.2009.0310 PMC2842802 19734187

[B28] Makarieva AM Gorshkov VG Li B-L Chown SL Reich PB Gavrilov VM Mean mass-specific metabolic rates are strikingly similar across life’s major domains: Evidence for life’s metabolic optimum Proc Nat Acad Sci USA 2008 105 16994 16999 10.1073/pnas.0802148105 18952839 PMC2572558

[B29] Hess B Boiteux A Krüger J Cooperation of glycolytic enzymes Adv Enzyme Regul 1969 7 149 167 4244004 10.1016/0065-2571(69)90016-8

[B30] Ingram DM Castleden WM Glucose increases experimentally induced colorectal cancer: A preliminary report Nutr Cancer 1981 2 150 152 10.1080/01635588109513676 7346780

[B31] Boubriak OA Urban JPG Cui Z Monitoring of metabolite gradients in tissue-engineered constructs Journal of the Royal Society Interface 2006 3 637 648 10.1098/rsif.2006.0118 PMC1664654 16971332

[B32] Murphy JB Hawkins JA Comparative studies on the metabolism of normal and malignant cells J Gen Physiol 1925 8 115 130 10.1085/jgp.8.2.115 19872184 PMC2140756

[B33] Vaupel P Mayer A Hypoxia in cancer: significance and impact on clinical outcome Cancer Metastasis Rev 2007 26 225 239 10.1007/s10555-007-9055-1 17440684

[B34] Ruan K Song G Ouyang G Role of hypoxia in the hallmarks of human cancer J Cell Biochem 2009 107 1053 1062 10.1002/jcb.22214 19479945

[B35] Brahimi-Horn MC Chiche J Pouyssegur J Hypoxia and cancer J Mol Med 2007 85 1301 1307 10.1007/s00109-007-0281-3 18026916

[B36] Russo CA Weber TK Volpe CM Stoler DL Petrelli NJ Rodriguez-Bigas M Burhans WC Anderson GR An anoxia inducible endonuclease and enhanced DNA breakage as contributors to genomic instability in cancer Cancer Res 1995 55 1122 1128 7866998

[B37] Meng AX Jalali F Cuddihy A Chan N Bindra RS Glazer PM Bristow RG Hypoxia down-regulates DNA double strand break repair gene expression in prostate cancer cells Radiotherapy and Oncology 2005 76 168 176 10.1016/j.radonc.2005.06.025 16026872

[B38] Rodriguez-Jimenez FJ Moreno-Manzano V Lucas-Dominguez R Sanchez-Puelles JM Hypoxia causes downregulation of mismatch repair system and genomic instability in stem cells Stem Cells 2008 26 2052 2062 10.1634/stemcells.2007-1016 18511603

[B39] Goldbeter A Berridge J Biochemical Oscillations and Cellular Rhythms: The Molecular Bases of Periodic and Chaotic Behaviour 1997 Cambridge: Cambridge University Press

[B40] Pye EK Menaker M Periodicities in the intermediary metabolism Biochronometry 1971 Washington, D.C: National Academy of Sciences 623 636

[B41] Sharma V Annila A Natural process–natural selection Biophys Chem 2007 127 123 128 10.1016/j.bpc.2007.01.005 17289252

[B42] Aromolaran AS Zima AV Blatter LA Role of glycolytically generated ATP for CaMKII-mediated regulation of intracellular Ca^2+^ signaling in bovine vascular endothelial cells American Journal of Physiology Cell 2007 293 C106 C118 10.1152/ajpcell.00543.2006 17344311

[B43] Yang J-H Yang L Qu Z Weiss JN Glycolytic oscillations in isolated rabbit ventricular myocytes J Biol Chem 2008 283 36321 36327 10.1074/jbc.M804794200 18948270 PMC2606010

[B44] Hilborn R Chaos and Nonlinear Dynamics: An Introduction for Scientists and Engineers 2001 New York: Oxford University Press

[B45] Badii R Politi A Complexity: Hierarchical Structures and Scaling in Physics 1999 Cambridge: Cambridge University Press

[B46] Tyner KM Kopelman R Philbert MA “Nanosized voltmeter” enables cellular-wide electric field mapping Biophys J 2007 93 1163 1174 10.1529/biophysj.106.092452 17513359 PMC1929021

[B47] Pokorny J Biophysical cancer transformation pathway Electromagn Biol Med 2009 28 105 123 10.1080/15368370802711615 19811394

[B48] Thompson JMT Stewart HB Nonlinear Dynamics and Chaos 2002 New York: John Wiley & Sons

[B49] May RM Simple mathematical models with very complicated dynamics Nature 1976 261 459 467 10.1038/261459a0 934280

[B50] Boiteux A Goldbeter A Hess B Control of oscillating glycolysis of yeast by stochastic, periodic, and steady source of substrate: a model and experimental study Proc Nat Acad Sci USA 1975 72 3829 3833 10.1073/pnas.72.10.3829 172886 PMC433089

[B51] Markus M Kuschmitz D Hess B Chaotic dynamics in yeast glycolysis under periodic substrate input flux FEBS Lett 1984 172 235 238 10.1016/0014-5793(84)81132-1 6235124

[B52] Martinez de la Fuente I Martinez L Veguillas J Dynamic behavior in glycolytic oscillations with phase shifts Biosystems 1995 35 1 13 10.1016/0303-2647(94)01473-K 7772719

[B53] Martinez de la Fuente I Martinez L Veguillas J Aguirregabiria JM Quasiperiodicity route to chaos in a biochemical system Biophys J 1996 71 2375 2379 10.1016/S0006-3495(96)79431-6 8913578 PMC1233727

[B54] Rietman EA Molecular Engineering of Nanosystems 2001 New York: Springer

[B55] Gurel O Gurel D Oscillations in Chemical Reactions 1983 New York: Springer-Verlag

[B56] Kaneko K Theory and applications of coupled map lattices 1993 New York: John Wiley & Sons

[B57] Fischer KH Hertz JA Spin Glasses 1993 Cambridge: Cambridge University Press

[B58] Sinha S Ditto WL Computing with distributed chaos Physical review E, Statistical physics, plasmas, fluids, and related interdisciplinary topics 1999 60 363 377 11969770 10.1103/physreve.60.363

[B59] von Klitzing L Betz A Metabolic control in flow systems. I. Sustained glycolytic oscillations in yeast suspension under continual substrate infusion Arch Mikrobiol 1970 71 220 225 10.1007/BF00410155 4319266

[B60] Ramanujan VK Herman BA Nonlinear scaling analysis of glucose metabolism in normal and cancer cells J Biomed Opt 2008 13 031219 10.1117/1.2928154 18601543

[B61] Aon MA Cortassa S O’Rourke B Percolation and criticality in a mitochondrial network Proc Nat Acad Sci USA 2004 101 4447 4452 10.1073/pnas.0307156101 15070738 PMC384767

[B62] Pikovsky A Rosenblum M Kurths J Synchronization: A Universal Concept in Nonlinear Sciences 2003 Cambridge: Cambridge University Press

[B63] Tinoco I Sauer K Wang JC Physical chemistry: principles and applications in biological sciences 1985 Englewood Cliffs, NJ: Prentice-Hall

[B64] Palsson BO Systems Biology: Properties of Reconstructed Networks 2006 Cambridge: Cambridge University Press

[B65] Hynne F Danø S Sørensen PG Full-scale model of glycolysis in Saccharomyces cerevisiae Biophys Chem 2001 94 121 163 10.1016/S0301-4622(01)00229-0 11744196

[B66] Steuer R Gross T Selbig J Blasius B Structural kinetic modeling of metabolic networks Proc Nat Acad Sci USA 2006 103 11868 11873 10.1073/pnas.0600013103 16880395 PMC1524928

[B67] Wolf J Passarge J Somsen OJ Snoep JL Heinrich R Westerhoff HV Transduction of intracellular and intercellular dynamics in yeast glycolytic oscillations Biophys J 2000 78 1145 1153 10.1016/S0006-3495(00)76672-0 10692304 PMC1300717

[B68] Gehrmann E Glasser C Jin Y Sendhoff B Drossel B Hamacher K Robustness of glycolysis in yeast to internal and external noise Phys Rev E Stat Nonlinear Soft Matter Phys 2011 84 021913 10.1103/PhysRevE.84.021913 21929026

[B69] Ko YH Smith BL Wang Y Pomper MG Rini DA Torbenson MS Hullihen J Pedersen PL Advanced cancers: eradication in all cases using 3-bromopyruvate therapy to deplete ATP Biochem Biophys Res Commun 2004 324 269 275 10.1016/j.bbrc.2004.09.047 15465013

[B70] Mathupala SP Ko YH Pedersen PL The pivotal roles of mitochondria in cancer: Warburg and beyond and encouraging prospects for effective therapies Biochim Biophys Acta 2010 1797 1225 1230 10.1016/j.bbabio.2010.03.025 20381449 PMC2890051

[B71] Morita T Watanabe Y Takeda K Okumura K Effects of pH in the in vitro chromosomal aberration test Mutat Res 1989 225 55 60 10.1016/0165-7992(89)90033-X 2913491

[B72] Morita T Nagaki T Fukuda I Okumura K Clastogenicity of low pH to various cultured mammalian cells Mutat Res 1992 268 297 305 10.1016/0027-5107(92)90235-T 1379335

[B73] Cifone MA Myhr B Eiche A Bolcsfoldi G Effect of pH shifts on the mutant frequency at the thymidine kinase locus in mouse lymphoma L5178Y TK^+/−^ cells Mutat Res 1987 189 39 46 10.1016/0165-1218(87)90031-0 3114628

[B74] Brusick D Genotoxic effects in cultured mammalian cells produced by low pH treatment conditions and increased ion concentrations Environ Mutagen 1986 8 879 886 10.1002/em.2860080611 3780620

[B75] Cipollaro M Corsale G Esposito A Ragucci E Staiano N Giordano GG Pagano G Sublethal pH decrease may cause genetic damage to eukaryotic cell: a study on sea urchins and Salmonella typhimurium Teratog Carcinog Mutagen 1986 6 275 287 10.1002/tcm.1770060404 2875539

[B76] Yuan J Narayanan L Rockwell S Glazer PM Diminished DNA repair and elevated mutagenesis in mammalian cells exposed to hypoxia and low pH Cancer Res 2000 60 4372 4376 10969780

[B77] Kondo A Safaei R Mishima M Niedner H Lin X Howell SB Hypoxia-induced enrichment and mutagenesis of cells that have lost DNA mismatch repair Cancer Res 2001 61 7603 7607 11606400

[B78] Suresh S Biomechanics and biophysics of cancer cells Acta Biomater 2007 3 413 438 10.1016/j.actbio.2007.04.002 17540628 PMC2917191

[B79] Ketene AN The AFM study of ovarian cell structural mechanics in the progression of cancer Masters thesis 2011 : Blacksburg, Virginia Virginia Tech, Mechanical Engineering

[B80] Creekmore AL Silkworth WT Cimini D Jensen RV Roberts PC Schmelz EM Changes in gene expression and cellular architecture in an ovarian cancer progression model PLoS One 2011 6 e17676 10.1371/journal.pone.0017676 21390237 PMC3048403

[B81] Fygenson D Braun E Libchaber A Phase diagram of microtubules Phys Rev E Stat Phys Plasmas Fluids Relat Interdiscip Topics 1994 50 1579 1588 10.1103/PhysRevE.50.1579 9962129

[B82] Sept D Xu J Pollard TD McCammon JA Annealing accounts for the length of actin filaments formed by spontaneous polymerization Biophys J 1999 77 2911 2919 10.1016/S0006-3495(99)77124-9 10585915 PMC1300564

[B83] Bolterauer H Limbach HJ Tuszyński JA Models of assembly and disassembly of individual microtubules: stochastic and averaged equations Journal of Biological Physics 1999 25 1 22 10.1023/A:1005159215657 23345684 PMC3456067

[B84] Desai A Mitchison TJ Microtubule polymerization dynamics Annu Rev Cell Dev Biol 1997 13 83 117 10.1146/annurev.cellbio.13.1.83 9442869

[B85] Pollard TD Rate constants for the reactions of ATP- and ADP-actin with the ends of actin filaments J Cell Biol 1986 103 2747 2754 10.1083/jcb.103.6.2747 3793756 PMC2114620

[B86] Bakhoum SF Thompson SL Manning AL Compton DA Genome stability is ensured by temporal control of kinetochore-microtubule dynamics Nat Cell Biol 2009 11 27 35 10.1038/ncb1809 19060894 PMC2614462

[B87] Thompson SL Bakhoum SF Compton DA Mechanisms of chromosomal instability Current biology 2010 20 R285 R295 10.1016/j.cub.2010.01.034 20334839 PMC3781365

[B88] Vitale I Galluzzi L Castedo M Kroemer G Mitotic catastrophe: a mechanism for avoiding genomic instability Nat Rev Mol Cell Biol 2011 12 385 392 21527953 10.1038/nrm3115

[B89] Crasta K Ganem NJ Dagher R Lantermann AB Ivanova EV Pan Y Nezi L Protopopov A Chowdhury D Pellman D DNA breaks and chromosome pulverization from errors in mitosis Nature 2012 482 53 58 10.1038/nature10802 22258507 PMC3271137

[B90] Zhang P Li H Tan X Chen L Wang S Association of metformin use with cancer incidence and mortality: A meta-analysis Cancer Epidemiol 2013 37 207 218 10.1016/j.canep.2012.12.009 23352629

[B91] Landman GWD Kleefstra N van Hateren KJJ Groenier KH Gans ROB Bilo HJG Metformin associated with lower cancer mortality in type 2 diabetes: ZODIAC-16 Diabetes Care 2010 33 322 326 10.2337/dc09-1380 19918015 PMC2809274

[B92] Li D Yeung S-CJ Hassan MM Konopleva M Abbruzzese JL Antidiabetic therapies affect risk of pancreatic cancer Gastroenterology 2009 137 482 488 10.1053/j.gastro.2009.04.013 19375425 PMC2735093

[B93] Pedersen PL Warburg, me and Hexokinase 2: Multiple discoveries of key molecular events underlying one of cancers’ most common phenotypes, the “Warburg Effect”, i.e., elevated glycolysis in the presence of oxygen J Bioenerg Biomembr 2007 39 211 222 10.1007/s10863-007-9094-x 17879147

[B94] Ko YH Verhoeven HA Lee MJ Corbin DJ Vogl TJ Pedersen PL A translational study “case report” on the small molecule “energy blocker” 3-bromopyruvate (3BP) as a potent anticancer agent: from bench side to bedside J Bioenerg Biomembr 2012 44 163 170 10.1007/s10863-012-9417-4 22328020

[B95] Ganapathy-Kanniappan S Geschwind J-FH Kunjithapatham R Buijs M Vossen JA Tchernyshyov I Cole RN Syed LH Rao PP Ota S Vali M Glyceraldehyde-3-phosphate dehydrogenase (GAPDH) is pyruvylated during 3-bromopyruvate mediated cancer cell death Anticancer Res 2009 29 4909 4918 20044597 PMC3743725

